# The Pilin Protein FimP from *Actinomyces oris*: Crystal Structure and Sequence Analyses

**DOI:** 10.1371/journal.pone.0048364

**Published:** 2012-10-31

**Authors:** Karina Persson, Anders Esberg, Rolf Claesson, Nicklas Strömberg

**Affiliations:** 1 Department of Chemistry, Umeå University, Umeå, Sweden; 2 Department of Odontology/Cariology, Umeå University, Umeå, Sweden; University of Texas-Huston Medical School, United States of America

## Abstract

The *Actinomyces oris* type-1 pili are important for the initial formation of dental plaque by binding to salivary proteins that adhere to the tooth surface. Here we present the X-ray structure of FimP, the protein that is polymerized into the type-1 pilus stalk, assisted by a pili-specific sortase. FimP consists of three tandem IgG-like domains. The middle and C-terminal domains contain one autocatalyzed intramolecular isopeptide bond each, a feature used by Gram-positive bacteria for stabilization of surface proteins. While the N-terminal domain harbours all the residues necessary for forming an isopeptide bond, no such bond is observed in the crystal structure of this unpolymerized form of FimP. The monomer is further stabilized by one disulfide bond each in the N- and C-terminal domains as well as by a metal-coordinated loop protruding from the C-terminal domain. A lysine, predicted to be crucial for FimP polymerization by covalent attachment to a threonine from another subunit, is located at the rim of a groove lined with conserved residues. The groove may function as a docking site for the sortase-FimP complex. We also present sequence analyses performed on the genes encoding FimP as well as the related FimA, obtained from clinical isolates.

## Introduction

Pili are hair-like organelles on the surface of bacteria. They are essential for functions such as biofilm formation, host-pathogen interactions and attachment to surfaces. Gram-negative pili are well studied both regarding structure and function [1], [2] whereas less is known about the structure, function and bioassembly of Gram-positive pili, even though they were first described decades ago [3]. However, during the last few years Gram-positive pilin structures from *C. diphtheriae* [4], *Actinomyces oris* [5], *Streptococcus pyogenes* [6], [7], [8], *Streptococcus pneumoniae* [9], [10], [11], [12], *Streptococcus agalactiae* [13], [14] and *Bacillus cereus* [15] have been described. In short the Gram-positive pili are built up from multiple copies of covalently linked major pilin proteins, resulting in a shaft. In addition, some pili, but not all, have minor pilin proteins incorporated into the stalk. In general, an adhesin is positioned at the tip. The recent advances in structure and function of Gram-positive pili are excellently reviewed by Kang and Baker [16].

Gram-positive proteins that function as building blocks for pili polymerization share some common characteristics. There is a signal peptide located in the N-terminus and an LPXTG motif in the C-terminus, followed by a transmembrane segment. The LPXTG motif is a sorting signal recognized by a sortase (a cysteine transpeptidase) that cleaves the protein between the threonine and the glycine in the motif. In the next step the threonine is covalently attached either to the cell-wall peptidoglycan if the sortase is a housekeeping sortase or to a lysine of a central pilin motif (WXXXVXVYPK) [17] of an identical pilin protein if a polymerization reaction is being catalyzed. The covalent polymerization of pilin proteins is performed by pili-specific sortases. The mechanism underlying the incorporation of auxiliary proteins into the fimbria is still not fully understood [18], [19], [20].

Dental plaque is a microbial biofilm built up from several hundreds of different bacterial species [21]. *Actinomyces spp* together with streptococci are among the first colonizers of the oral biofilm and promote further biofilm formation by their interaction with a wide variety of proteins and carbohydrates on microorganisms and host cells, or from saliva. *A. oris* (previously *Actinomyces naeslundii* genospecies 2 [22]) can express two different types of pili: type-1 and type-2. Type-1 pili mediate the first attachment to host salivary proline-rich proteins (PRPs) that coat the tooth, whereas type-2 pili mediate attachment to carbohydrate structures on oral streptococci [23], [24] and host cells [25]. The two types of pili are encoded by two separate gene clusters. Each gene cluster contains three genes that encode a large putative adhesin, the pilus shaft protein and the pili-specific sortase. The encoded pilin proteins are as follows: FimQ, FimP and SrtC-1 for type-1 and FimA, FimB and SrtC-2 for type-2 [26], [27]. The pilus shaft proteins FimP and FimA are 28% identical in sequence and are very similar in size. The sortases SrtC-1 and SrtC-2 share 42% sequence identity within the enzymatic domain. In contrast, the putative adhesins differ in both size and sequence (1413 residues for FimQ and 976 residues for FimB). This may reflect their differences in binding specificity.

Intriguingly, it was recently shown for type-2 pili that the pili stalk alone (FimA) is involved in the co-aggregation reaction with carbohydrates [28] which leaves the function of FimB unclear. However, in a similar study on the type-1 pili it was shown that the presumed adhesin, FimQ, did indeed interact with PRPs and that the shaft protein FimP appeared not to be involved in this interaction [29]. To unravel some of the basics of the molecular function of these pili it is necessary to study the molecular organization of the participating proteins. Recently the crystal structure of the carboxy-terminal fragment of *A. oris* FimA was presented [5] as well as the crystal structure of the FimP-specific sortase SrtC-1 [30]. To gain more insight into the structure and function of the *A. oris* type-1 pili, we have solved the structure of the FimP shaft protein, refined to 1.6 Å resolution and analyzed the conserved and polymorphic FimP and FimA amino acid variations among clinical isolates.

## Results and Discussion

### Structure Determination

**Figure 1:**
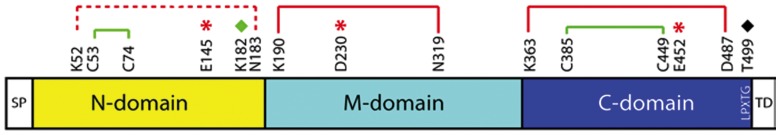
Domain architecture of FimP. The FimP protein is comprised of a signal peptide (SP), an N-terminal domain, a middle domain and a C-terminal domain followed by an LPXTG motif and a transmembrane domain (TD). Residues involved in isopeptide and disulfide bonds are illustrated with bars and stars, in red and green, respectively. A lysine and a threonine involved in pili polymerization are illustrated with a green and black diamond respectively.

A construct comprising residues 31–491 of FimP (FimP_31–491_) from *A. oris* strain T14V was expressed in *E. coli*, purified and crystallized. The N-terminal signal peptide, the C-terminal transmembrane helix and the cell-wall anchoring motif LPLTG were not included in the construct (Fig. 1). Phases were experimentally determined using single wavelength anomalous dispersion (SAD) of a selenomethionine (SeMet) labeled triple mutant, FimP-3M, in which three isoleucines (Ile-121, Ile-204 and Ile-347) were exchanged for methionines [31]. SAD data were collected to 2.0 Å resolution and an initial model was built. The model was further refined against a native data set to 1.6 Å. The asymmetric unit contains one molecule of FimP_31–491_. The final model is well ordered with an overall B-factor of 18.7 Å^2^ (Table 1). The refined model comprises residues 35–490. No or weak electron density was observed for the loop residues 57–63 and 70–72. In addition, four metal ions and 833 water molecules were included.

**Table 1:** Data collection, refinement and model quality statistics for FimP.

	Native FimP	SeMet FimP-3M
**Data collection**		
Space group	*P*2_1_2_1_2	*P*2_1_2_1_2
Cell dimensions a, b, c(Å)	77.24, 176.59, 40.12	76.27, 168.13, 39.76
Wavelength (Å)	0.9334	0.97918
Resolution (Å)	46.82–1.6	45.16–2.0
Highest resolution shell(Å)	1.69–1.6	2.11–2.0
Total reflections^a^	382619 (27906)	220706 (6864)
Unique reflections^a^	71266 (9922)	35477 (1257)
I/σ (I)^a^	21.4 (5.8)	29.9 (15.7)
R_sym_(%)a, b	5.9 (16.1)	4.0 (9.2)
Completeness (%)^a^	97.5 (95.1)	99.8 (99.4)
Overall redundancy	5.4 (2.8)	6.2 (6.3)
**Refinement**		
No. reflections in working set	67601	
No. reflections in test set	3593	
Rwork/Rfree (%)^c^	16.93/19.57	
Average B-factors (Å^2^)		
Wilson plot	20.4	
Protein	18.7	
Water	30.3	
Metal ions	22.1	
No. protein atoms	3419	
No. metal ions	4	
No. water molecules	833	
RMSD from ideal		
Bond lengths (Å)	0.12	
Bond angles (°)	1.432	
Ramachandran plot		
Preferred, allowed,outliers (%)	95.9/3.2/0.9	

a Values in parentheses indicate statistics for the highest resolution shell.

b *R*_sym_ = Σ*_hkl_* Σ*_i_* |*I*_i_ *(hkl)*−<*I*(*hkl*)>|/Σ*_hkl_* Σ*_i_ I*_i_ (*hkl*), where *I_i_*(*hkl*) is the intensity of the *i*th observation of reflection *hkl and <I(hkl)>* is the average over of all observations of reflection *hkl.*

c *R*_work_ = Σ | |*F*_obs_|−| *F*_calc_| |/Σ | *F*_obs_|, where *F*_obs_ and *F*_calc_ are the observed and calculated structure factor amplitudes, respectively. *R*_free_ is *R*_work_ calculated using 5% of the data, randomly omitted from refinement.

### Overall Structure of FimP

**Figure 2:**
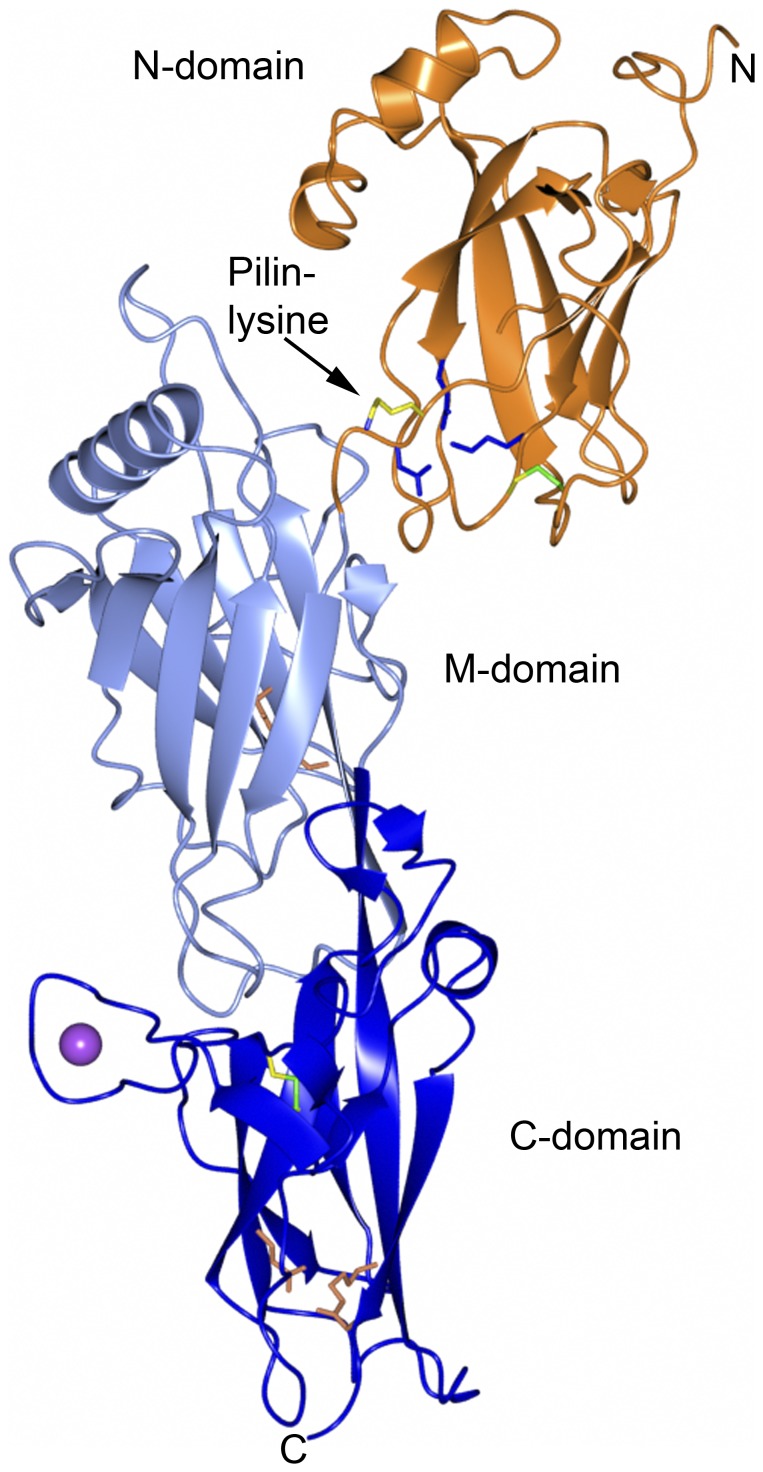
Overall structure of FimP. Ribbon representation of FimP_31–491_. The N-, M- and C-domains are in coral, light blue, and blue respectively. The residues forming isopeptide bonds in the M- and C-domains are shown as coral stick models and the disulfides in the N- and C-domains as green sticks. The lysine in the pilin motif is shown as a stick model in yellow and blue. The Ca^2+^ ion bound to the C domain is depicted as a sphere in magenta. Residues in the N-domain putatively involved in isopeptide bond formation are shown in blue. N- and C-termini are indicated.

FimP is an elongated protein, approximately 105 Å long and 35 Å wide, folded into three IgG-like domains: the N-terminal (N), middle (M) and C-terminal (C) domains (Fig. 2). The IgG-folds are of the CnaA- (M-domain) or the CnaB- (the N- and C-terminal domains) types. These IgG-like folds are extensively found in cell surface adhesins [32]. The M-domain (187–355) and the C-domain (356–490) are rigidly connected in line via a shared strand whereas the N-domain (35–186) and M-domain are connected via a hinge. The mobility of the hinge is reflected by the slight alternation in N-domain position, observed when comparing the native structure and the SeMet-labeled FimP-3M structures. The difference in N-domain rotation is also reflected by the difference in unit cell dimensions, where the b-axis is approximately 5% shorter in the SeMet structure than in the native structure. The shift in the N-domain positions may be caused by one of the introduced (seleno)methionines, I347M. Residue 347 is located at the interface between the domains and a change from isoleucine to selenomethionine can indeed alter the interaction properties. In the FimP-3M structure 165 contacts are observed between the N- and M-domains, compared to 213 in the native structure as calculated with the program CONTACTS in the CCP4 suite [33]. The selenomethionine itself at position 347 has 20 contacts whereas the native isoleucine has 39. The other mutants, I121M and I347M, do not cause any changes.

**Figure 3:**
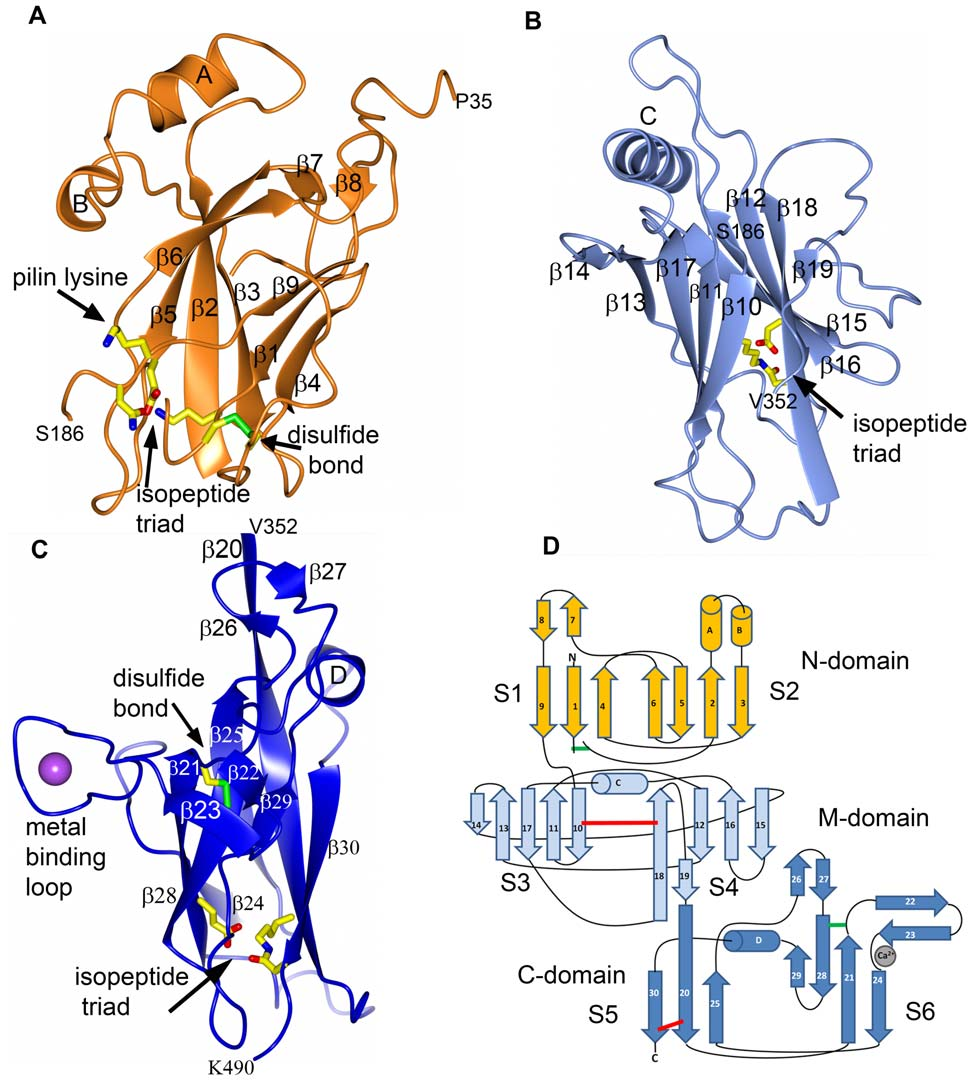
Domains and topology diagram. Each domain (N-, M- and C-) with labeled secondary structure. **A:** N-domain in coral, **B:** M-domain in light blue, **C:** C-domain in blue. The Ca^2+^ ion is depicted as a magenta sphere. **D:** Topology diagram of FimP with domains colored as in **A**–**C**. The isopeptides are depicted as red, and disulfides as green, bars. The Ca^2+^ ion coordinated by the loop is shown as a grey sphere.

The N-domain comprises a β-sandwich of one three-stranded, mixed β-sheet (S1) and one four-stranded (S2) anti-parallel sheet. Two helices (HA and HB) pack against the S2 sheet. In addition, two short anti-parallel β-strands, β7-β8, connected by a long loop, are located perpendicular to the β-sandwich (Fig. 3a and 3d).

The M-domain comprises a β-sandwich of two five-stranded anti-parallel sheets, S3 and S4. A helix (HC) is wedged in between the upper part of the sandwich. Below the S4 sheet two anti-parallel strands, β26 and β27, are located. A 20-residue long loop that connects β18 with β19 packs against the S4 sheet. The continuation of β19, β20 connects the M- and C-domains (Fig. 3b and 3d).

The C-domain, like the N-domain, consists of a β-sandwich of one mixed, three-stranded and one four-stranded β-sheet, S5 and S6, respectively. A short helix (HD), is located at the top of the S5 sheet. A long segment connects strands β21 and β24 and contains a β-hairpin (β22/β23) packed against the S6 sheet, as well as a long loop that coordinates a Ca^2+^ ion (Fig. 3c and 3d).

### All Three FimP Domains are Stabilized with Covalent Bonds

The presence of intramolecular isopeptide bonds in Gram-positive surface proteins was first described for the *S. pyogenes* pilin Spy0128 [6] and these bonds are now considered a widespread feature among Gram-positive surface proteins. Intramolecular isopeptide bonds are used for increasing the stability of the surface exposed protein, both regarding the sensitivity to proteases and mechanical force. Generally, a covalent amide bond is formed between the NZ atom of a lysine and the CG atom of an asparagine or an aspartic acid, assisted by the presence of a close acidic residue in an hydrophobic environment.

**Figure 4:**
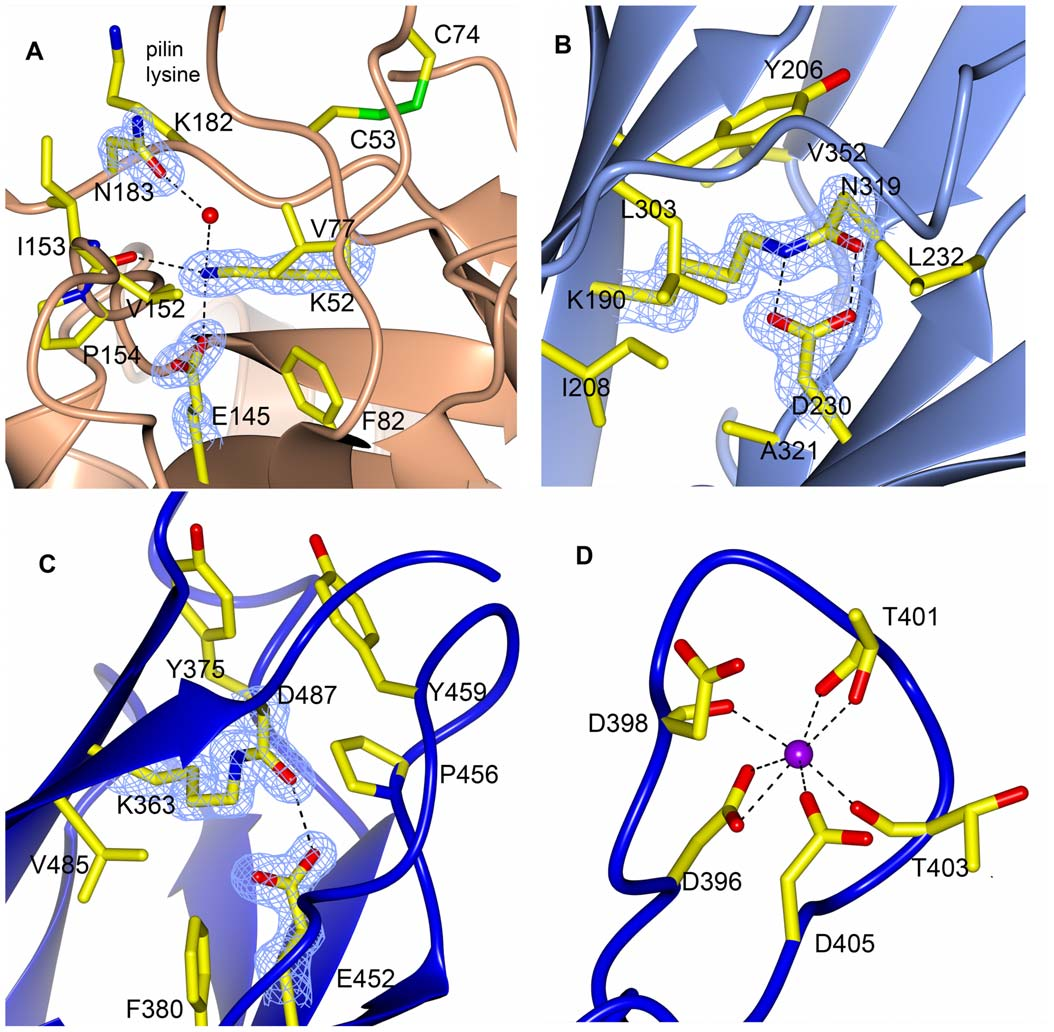
Stabilizing isopeptide bonds (formed and unformed) and a metal binding loop. **A:** The putative isopeptide residues in the N-domain, Lys-52, Asn-183 and Glu-145, do not form an isopeptide bond in the crystal structure. **B**: The M-domain isopeptide bond formed between Lys-190 and Asn-319 with the catalytic Asp-230. Asp-230 forms a bidentate hydrogen bond with the isopeptide bond. **C:** The C-domain isopeptide bond formed between Lys-363 and Asp-487 with the catalytic Glu-452. Glu-452 forms one hydrogen bond with the isopeptide bond carbonyl oxygen. **D:** A Ca^2+^ ion is coordinated by five residues of a loop that protrudes from the C-domain. Residues involved in isopeptide bond formation are represented as stick models, colored by atom type in a simulated annealing, omit Fo-Fc maps contoured at 4σ. Hydrogen bonds are shown as broken lines. Surrounding hydrophobic residues are shown as stick models.

Accordingly, isopeptide bonds are also found in FimP_31–491_. The M-domain is stabilized by a bond between Lys-190 and Asn-319, linking two strands that run anti-parallel to each other (Fig. 4b). The strands originate from the S3 and S4 sheets respectively. The formation of the isopeptide bond is catalyzed by Asp-230 located in the S4 sheet. Asp-230 forms bidentate hydrogen bonds with the amide hydrogen and the carbonyl oxygen of the isopeptide bond. The linkage stacks with the aromatic Tyr-206 and is surrounded by additional hydrophobic residues (Ile-208, Leu-303, Ala-321, Leu-232 and Val-352).

The C-domain also contains one isopeptide bond, formed between Lys-363 and Asp-487 (Fig. 4c). In this domain the bond links the first and last β-strands of the domain, β20 and β30, that run parallel to each other in the S5 sheet. The formation of the bond is assisted by the presence of Glu-452, previously described as the E-box motif. The glutamic acid forms only one hydrogen bond to the carbonyl oxygen of the isopeptide crosslink. Similar to the M-domain, the bond is surrounded by aromatic and hydrophobic residues, such as Tyr-375, Phe-380, Pro-456, Tyr-459 and Val-485.

There is no isopeptide bond observed in the N-domain but when superimposing the C-domain with the N-domain it is obvious that the N-domain also has all the necessary amino acids, Lys-52, Asn-183 and Glu-145, to form an isopeptide linkage. However, in our crystal structure, Asn-183 is located on the hinge between the N- and M-domains, and its CG atom is too far away from the lysine NZ atom for a bond to form, although the two side chains are connected via a water molecule (Fig. 4a). Instead the residue following the lysine, Cys-53, forms a disulfide bridge with Cys-74, thereby contributing to the stability of the S1 sheet by other means. If formed, the isopeptide bond in the N-domain would link the two parallel strands of the three-stranded sheet, similar to the bond in the C-domain. Interestingly, the Asn-183 residue expected to form the isopeptide bond in the N-domain, is positioned next to Lys-182, the lysine that is crucial for formation of the *inter*molecular bond with Thr-499 of another FimP subunit upon pili polymerization.

The presence of the two isopeptide bonds was evident from the continuous electron density between the lysines and the asparagine (in the M-domain) or the aspartic acid (in the C-domain). Their existence was also confirmed by ESI-TOF-MS. Site directed mutagenesis was applied to generate two mutants, D230A and E452A, each replacing the catalytic residue of the isopeptide triads with alanine. Mass analyses of the mutant and wild type proteins confirmed the approximate mass losses of NH_3_ (M-domain), H_2_O (C-domain) or both (wild type) upon isopeptide bond formation compared to the masses calculated from the primary FimP sequence (Table 2). Thus the mutants, where the respective catalytic residue has been removed, form one isopeptide bond, whereas in the native protein two isopeptide bonds are formed. Accordingly, the ESI-TOF-MS experiments also verify that a third isopeptide bond is not present in the unpolymerized recombinant form.

**Table 2:** Mass determination of wild type and mutant FimP_31–491_ proteins.

Protein	Average mass (Da)		Difference (Da)
	Calculated from sequence	Observed by MS^a^	calculated-observed
FimP_31–491_	53067	53033	−34
FimP_31–491_ D230A	53023	53006	−17
FimP_31–491_ E452A	53009	52993	−16

a MS, ESI-TOF mass spectrometry.

Not all Gram-positive bacteria use disulfide bonds to stabilize secreted proteins although it is a common tool used by Actinobacteria [34]. In agreement with that, we observe that the FimP shaft protein is stabilized by two disulfide bonds, one in the N-domain and one in the C-domain, the domains that share the CnaB fold. In the N-domain, C53 and C74 form a disulfide bond between the start and the end of loop β1-β2, of which the C53 is directly positioned after the lysine putatively involved in isopeptide bond formation, as discussed above (Fig. 3a,d 4a). The loop segment connected by the disulfide is the most flexible in the FimP_31–491_ structure and interpretable electron density is missing for residues 57–63 and 70–72.

In the C-domain a disulfide bond between C385 and C449 joins the S6 sheet of the β-sandwich and the β22-β23 β-hairpin, the structural segment that is followed by the protruding metal-coordinating loop. (Fig. 3c, d).

### Metal-binding Sites

Four metal ions are found in the FimP_31–491_ structure and they are modeled as calcium due to the high concentration of calcium in the crystallization conditions. Three of them are likely to be present due to the crystallization solution; one is located between symmetry-related molecules and the other two are coordinated by only three protein atoms each. The fourth metal seems to have a structural role and is coordinated by a long loop in the C-domain, protruding from the S6 sheet. This metal is coordinated by seven oxygen atoms: Asp-396 (OD1 and OD2), Asp-398 (O), Thr-401 (OG1 and O), Thr-403 (O) and Asp-405 (OD2) (Figure 4d). The distances between the metal and the seven coordinating oxygen atoms in the loop refine to an average of 2.43 Å which is more consistent with Ca^2+^ (2.33–2.39Å) than to for instance Mg^2+^ (2.05–2.26Å) [35]. Moreover the metal coordinated by the loop refines to a B-factor of 23.5 Å^2^ with is similar to the surrounding oxygen atoms that are refined to an average of 26.4 Å^2^.

### Comparison with FimA and Other Pilin Structures

A structure similarity search of the FimP_31–491_ structure in the Protein Data Bank using the DALI server [36] found several Gram-positive surface proteins as structural relatives. SpaA from *C. diphteriae* (PDB 3HR6, Z-score of 24.8 [4]), which also contains three IgG-like domains, was identified as the closest structural relative. Separate searches performed with the N-domain alone and with the M- and C-domains together gave the same hit. The N-domain search resulted in a Z-score of 14.9 and an root mean square deviation (RMSD) of 1.9 Å on 115 aligned Cα residues compared to SpaA. When the M- and C-domains were used, the Z-score was 21.6 and the RMSD 3.3 Å on 266 aligned Cα atoms. SpaA and and FimP_31–491_ are very similar in structure and share the same topology of the β-sheets. The SpaA and the FimP_31–491_ structures also share the positions of the isopeptide bonds in the M- and C-domains. The differences between the two structures are mainly the positions of the bound metal ions. Their respective structural metal ion is located in similar, but not identical, areas of the molecules close to the interface between the M- and C-domains, but coordinated by loops from different domains. In FimP, the metal-coordinating loop belongs to the C-domain and protrudes from the core of the molecule, whereas the metal-binding loop in SpaA originates from the M-domain and is more involved in domain-domain interactions. Both FimP and SpaA have a disulfide bond in their respective C-domain, located in similar positions, however the presence of a disulfide bond in the N-domain is unique for FimP. Neither FimP nor SpaA has an isopeptide bond in the N-domain, although FimP has all the residues needed to form such a bond and SpaA does not. Other DALI hits, with somewhat lower Z-scores, were the C-terminal fragment of FimA from *A. oris* (PDB code 3QDH, Z-score, 19.9 [5]) that could be superposed onto FimP_31–491_ with an RMSD of 2.8 Å on 235 aligned Cα atoms. Similarly, the pilus protein GBS80 from *S. agalactiae* can be superimposed on the FimP with an RMSD of 3.2 Å on 246 aligned Cα atoms (PDB code 3PF2, Z-score 15.9 [13]). These are all Gram-positive pilin proteins consisting of three IgG-like domain (only M- and C domains are present in the models of FimA and GBS80).

### The Pilin Motif is Located in a Groove

**Figure 5:**
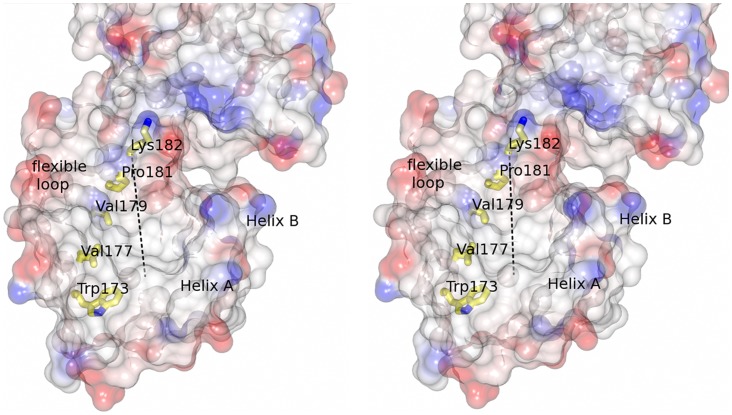
The pilin motif forms a groove. Stereo representation of the pilin motif residues lining a cleft that runs through the N-domain. Lys-182, involved in polymerization of FimP subunits, is localized at the rim of the cleft. The domain is presented as a semi-transparent electrostatic surface, colored in red and blue according to negative and positive electrostatic potential, respectively. The residues in the pilin motif are shown as stick models. The groove is highlighted with a dashed line.

In FimP, the lysine of the pilin motif (WNYNVHVYPK) is exposed on the hinge that connects the N- and M-domains, and is thereby available for the sortase mediated pili polymerization reaction (Fig. 3a, 4a and 5). The pilin lysine, Lys-182, is located at the end of a mostly non-polar groove formed between helix A, the β-sandwich, and the mobile loop of the N-domain. The groove is lined by the pilin-motif conserved residues Trp-73, Val-177, Val-179, and Pro-181 with the pilin lysine Lys-182 located at the rim of the channel. The conserved Tyr-180 stacks with His-51 and forms a hydrogen bond with the main chain amide of Asp-69, located close to the mobile loop. The isopeptide triad (Lys-52, Asn-183, Glu-145) is positioned within the groove however the isopeptide bond is not observed in the crystal structure as discussed above.

The recent structures of the *S. pneumoniae* pilin RrgB give an explanation of the function of the N-domain cleft and what dictates its isopeptide bond formation. When RrgB was crystallized in a form devoid of the sorting motif, an isopeptide bond in the N-terminal domain was not formed [10]. However, when a longer RrgB form was crystallized, its sorting motif was shown to interact with the pilin lysine of a neighboring pilin leading to a conformational change in the N-terminal domain and the subsequent formation of the covalent isopeptide bond [11].

In concurrence with the RrgB structures we propose a similar role for the FimP N-terminal groove; that the cleft and mobile loop (residues 56–64) function as recognition and interaction sites for the sortase-FimP complex during pili polymerization and that a preformed isopeptide bond (juxtaposed to the pilin Lys-182) results in a protein too rigid to properly present the pilin lysine to the sortase. However, during pili polymerization more strain may be imposed on the flexible hinge between the N- and M-domains and Asn-183 and Lys-52 may come close enough to form a bond. Indeed, in the BcpA pilin the N-domain isopeptide bond is observed only after BcpA polymerization [15].

In the quest for new drug candidates against pili-bearing Gram-positive bacteria, the N-domain groove may constitute a target for the development of pili inhibiting peptides or chemicals, with the sorting signal as the original template.

### Structural Comparison with FimA

**Figure 6:**
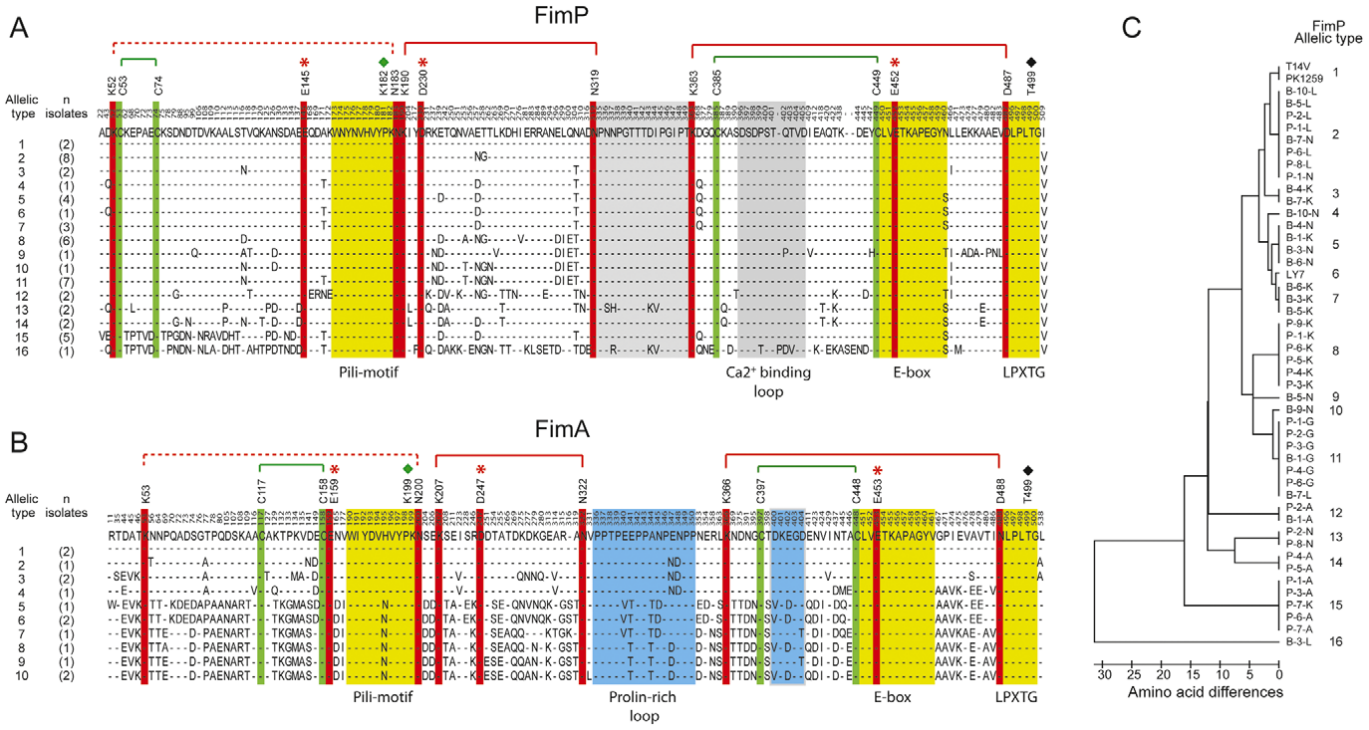
Sequence analyses of FimP and FimA among *A. oris* isolates. **A:** Sequence alignment of FimP (n = 48) with fully conserved isopeptide bond triads (red), disulfide bonds (green), a conserved metal binding loop (grey) and pilin-, E-box- and LPLTG motifs in yellow. **B:** Sequence alignment of FimA (n = 14) with fully conserved isopeptide bond triads (red), disulfide bonds (green), a conserved proline-rich loop (blue) and pilin-, E-box- and LPLTG motifs in yellow. In addition, in A and B, polymorphic amino acid residues are shown (single letter codes). The top lines represent the consensus sequence and amino acid positions based on FimP and FimA respectively of reference strain T14V. **C:** Neighboring joining tree with sixteen allelic or sequence *fimP* types among *A. oris* isolates (n = 48) due to the single amino acid variations.

*Actinomyces* spp can express two different forms of pili, encoded by separate gene clusters. The respective shaft proteins, FimP and FimA, are similar in terms of size and sequence. FimP consists of 533 residues and FimA of 535. A sequence analysis reveals that the major features; the pilin motif, the number of cysteines, the residues involved in isopeptide bond formation and the LPXTG motif are strictly conserved. Thus FimP and FimA are expected to have very similar structures. On the other hand, some differences are expected to be found, e.g., in the organization of loops and metal binding (Fig. 6).

Limited proteolysis experiments digesting FimA and FimP with trypsin gave different results which indicate some differences in exposure of certain regions (data not shown). Trypsin treatment of FimP generated a cut at Lys-62, located in the mobile loop of the N-domain whereas FimA (from *A. naeslundii* strain 12104) was cut at the pilin motif lysine as well as in the C-domain (Lys-372). Mishra *et al* [5] reported that FimA from *A. oris* strain T14V is similarly cut at the pilin lysine. This difference in sensitivity to trypsin implies that FimP is more rigid than FimA in which the pilin motif is more exposed.

**Figure 7:**
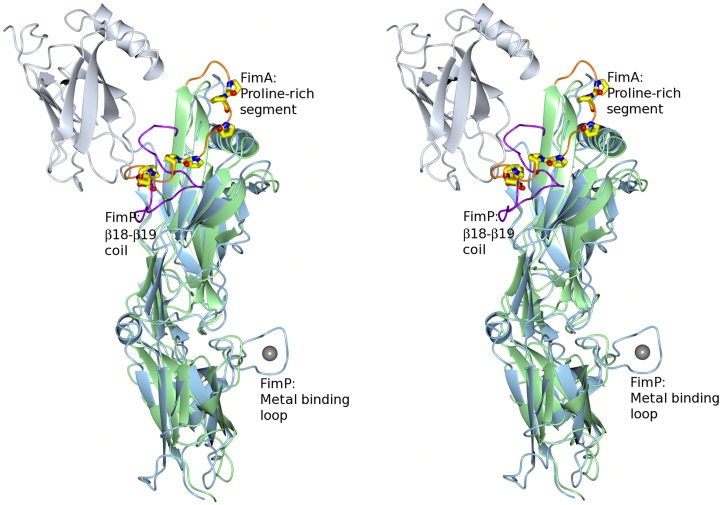
Superposition of FimP and FimA. The FimP crystal structure superimposed onto the corresponding structure of FimA (M- and C domains). The FimP structure is depicted in light blue and FimA in green. The proline-rich segment in FimA is coloured in orange with the prolines as stick models. The equivalent loop in FimP is colored in purple. The metal ion coordinated by a FimP loop is depicted in grey. The N-terminal domain of FimP is colored in light grey. The figure is shown in stereo.

A structural comparison between FimP_31–491_ and FimA (M- and C-domains) revealed that the major differences between the two structures are the loops connecting the β-strands. The most prominent difference is the segments that pack against the S4 sheet of their respective M-domains. In FimA this constitutes a proline-rich segment (_332_PPTPETPPTDPENPP_347_) that runs diagonally over half the sheet. In FimP the equivalent segment is a long, coiled region, located between β18 and β19, that folds over the whole S4 sheet like a clamp (Fig. 7).

Another distinct structural difference is the FimP metal-binding loop that protrudes from the C-domain like a knob. This loop has no equivalent in FimA. Such a significant structural element is likely to be available for recognition by certain microbes of the biofilm. In analogy, the surface adhesin SspB of the oral bacteria *Streptococcus gordonii* exhibits a surface-exposed helix, stabilized by a metal ion [37]. This helix is solely recognized by the periodontal pathogen *Porphyromonas gingivalis* [38]. Likewise, the proline-rich segment in FimA may function as a recognition site for other bacteria-bacteria interactions. However, to further unravel the complex mechanisms that control colonization and formation of the oral biofilm, extensive structural and functional studies have to be performed.

Sequence comparison of the FimP and FimA N-domains indicates different positions of the disulfide bridges. In FimP the first cysteine of the N-domain is located directly after the presumed isopeptide forming lysine and participate in a disulfide bond that unites the ends of a flexible loop. Also in FimA, the presumed disulfide bond is located near the putative isopeptide bond but with a different organization where its second cysteine is located directly before the catalytic glutamic acid. When the FimA sequence is applied on the FimP template, the first cysteine is located in the coil after the two helices A and B and the second in the S2 sheet, next to the isopeptide bond glutamic acid, as discussed. Assuming that a disulfide bridge is formed also in FimA, we suggest that its S2 sheet is more occluded due to tighter packing with the N-domain helices. The overall appearance of the N-domains may therefore be quite different, which indeed is reflected by their different sensitivity to trypsin. The structural differences are consistent with the differential functional roles of the two pili. The evolution and adaptation of type-1 (FimP) and type-2 (FimA) pili to different intraoral niches and tropisms, suggest their pilin proteins to be organized and function more differently than assumed from their co-presence in the *Actinomyces* genus. The metal binding loop, proline-rich motifs or differential N-domain structures could accordingly participate in various intergeneric bacteria-bacteria adhesion (co-aggregation) or host-bacteria adhesion partnerships. Notably, *Actinomyces* has multiple co-aggregation partners and hosts, therefore their pilin proteins are expected to possess multiple binding activities.

### Conserved or Polymorphic FimP and FimA Features among Clinical *A. oris* Isolates

Sequencing of the *fimP* gene from six *A. oris* reference strains (T14V, PK1259, P-1-N, P-8-L, LY7 and P-1-K) expressing FimP pili of defined binding profiles [39], [40] and clinical isolates (n = 42) revealed a highly conserved (97% identity/98% similarity) sequence (Fig. 6a). All three isopeptide bond triads, the cysteine bridges, pilin and LPLTG motifs were fully, and the metal binding loop highly, conserved among the strains (n = 48). The variable or polymorphic amino acid sites (19%), which localized generally over the domains, loops and β-strands without any apparent clustering or patterning, generated a total of sixteen allelic or sequence types (Fig. 6c).

FimP was also compared to FimA, deduced from *fimA* from *A. oris* isolates (n = 14). The FimP and FimA proteins showed 31% identity/45% similarity and fully conserved isopeptide bond triads, number of cysteines, pilin and LPLTG motifs. The metal binding loop was proved to be unique for FimP and the proline-rich segment unique for FimA. In addition, FimA from *A. oris* (n = 14) and *A. naeslundii* (n = 17) were highly related (64% identity/76% similarity) with fully conserved isopeptide bond triads, position of cysteines, pilin and LPLTG motifs as well as proline residues in the proline-rich segment. The sequence alignment of FimA from *A. oris* is presented in Fig. 6b.

## Materials and Methods

### Cloning, Purification and Crystallization

FimP_31–491_was cloned from *A. oris* strain T14V, expressed and crystallized as described [31]. In short, N-terminal 6His-tagged FimP was purified by nickel-affinity chromatography followed by size-exclusion chromatography. The protein was concentrated to 92 mg/ml in 20 mM Tris-HCl, pH 8.0. Selenomethionine (SeMet)-substituted protein was obtained after mutating three isoleucines to methionines (FimP-3M) [31]. The protein was subjected to *in situ* proteolysis with 1% (w/w) α-chymotrypsin immediately before crystallization set up. Crystals of the native protein were obtained in 100 mM sodium acetate pH 5.0, 100 mM CaCl_2_ and 20% PEG4000. Crystals of the SeMet containing protein were obtained in the same conditions after seeding with the native crystals.

### Generation of Isopeptide Bond Mutants

Generation of the mutants D230A and E452A was performed using the overlap extension PCR technique [41]. In short, for each mutant a first round of PCR generated two overlapping PCR fragments. In the second PCR step the two fragments were hybridized and amplified. The final PCR products were ligated into an expression vector as described [31]. The mutant proteins were purified as the native protein.

### Data Collection and Structure Determination

Crystals were flash-cooled in liquid nitrogen after a 30 s soak in the crystallization solution supplemented with 20% glycerol. X-ray diffraction data of the native crystals were collected at beamline ID14-1 and of the SeMet crystals at beamline ID-23 at the European Synchrotron Radiation Facility, ESRF, in Grenoble, France to 1.6 and 2.0 Å resolution respectively. Data were processed with XDS [42] and scaled with SCALA from the CCP4 program suit [33]. The SeMet containing structure was solved with SAD-phasing using AutoRickshaw [43]. Density modification and automatic model building were performed using AutoRickshaw and ArpWarp [44] and resulted in a readily interpretable map. For refinement, 5% of the reflections were removed for the calculation of Rfree. The model was further built using rounds of manual building in COOT [45] and refinement using phenix.refine [46]. The first refinement step included simulated annealing starting at 5000 K.

The native structure was solved with MOLREP [47] using the FimP SeMet structure as the search model. Due to movements of the N-domain, only residues 190–490 were placed in the molecular replacement solution and residues 35–189 were built manually. The native structure was refined as the SeMet structure. The quality of the model was analyzed with WHATCHECK [48]. Ramachandran statistics were obtained using COOT [45]. Crystallographic statistics are presented in Table 1.

The X-ray coordinates and structure factors have been deposited in the Protein Data Bank under accession codes 3UXF.

### Mass Spectrometry Analyses

Buffer solutions of FimP, FimP-D230A, and FimP-E452A were exchanged for water by dialysis. Accurate molecular masses were determined by ESI-TOF mass spectrometry at Proteomics Karolinska (PK) Institute, Stockholm, Sweden.

### Limited Proteolysis and N-terminal Sequencing of FimA and FimP Fragments

Purified recombinant FimP (*A. oris* strain T14V) and FimA (*A. naeslundii* strain 12104) in 20 mM CaCl_2_, 50 mM Tris-HCl pH 8.0, 1% glycerol and 2 mM DTT were incubated with trypsin at a ratio 1∶100 trypsin: FimP/FimA at 293 K for 1 h. The cleavage products were separated on by SDS-PAGE and transferred to a PVDF membrane. N-terminal sequencing of the cleavage products was performed.

### Sequencing of FimP and FimA from *A. oris* and *A. naeslundii* Strains

The *fimP* genes were sequenced from *A. oris* clinical isolates (n = 42) and reference strains (n = 6; T14V, PK1259, P-1-N, P-8-L, LY7 and P-1-K). The *fimP* genes were amplified by PCR using purified genomic DNA and the Ready-To-Go PCR polymerase kit (GE healthcare).

The *fimA* genes were sequenced from *A. oris* (n = 14) and *A. naeslundii* (n = 17) isolates. The *fimA* genes were amplified by PCR from purified DNA using the Iproof High-Fidelity enzyme (BioRad). Purified fragments were cloned into Zero Blunt® TOPO® PCR Cloning Kit (Invitrogen) and sequenced. All sequencing was performed by Eurofins MWG Operon. The *fimP* (corresponding to amino acids 31–516) and *fimA* (corresponding to the full length protein) sequences were assembled and analyzed using the CodonCode Aligner and MEGA5 software. Identity/similarity values were generated using the NCBI web site.
